# Migraine with active headache was associated with other painful physical symptoms at two-year follow-up among patients with major depressive disorder

**DOI:** 10.1371/journal.pone.0216108

**Published:** 2019-04-30

**Authors:** Ching-I Hung, Chia-Yih Liu, Ching-Hui Yang, Shuu-Jiun Wang

**Affiliations:** 1 Department of Psychiatry, Chang Gung Memorial Hospital at Linkou, Tao-Yuan, Taiwan; 2 School of Medicine, Chang Gung University, Tao-Yuan, Taiwan; 3 Department of Nursing, Mackay Medical College, New Taipei, Taiwan; 4 School of Medicine, National Yang-Ming University, Taipei, Taiwan; 5 Brain Research Center, National Yang-Ming University, Taipei, Taiwan; 6 Neurological Institute, Taipei Veterans General Hospital, Taipei, Taiwan; Public Library of Science, UNITED STATES

## Abstract

**Background:**

Few studies have investigated the associations of comorbid migraine with other painful physical symptoms (PPS) in patients with major depressive disorder (MDD) at the two-year follow-up point. This study aimed to investigate this issue.

**Methods:**

At baseline, 155 outpatients with MDD were enrolled. Migraine was diagnosed at baseline according to the *International Classification of Headache Disorders*. At follow-up, data of 101 subjects were analyzed. The average intensities of head, bone and/or joints, back, chest, abdomen, neck and/or shoulder, general muscle, and limb pain in the past week were evaluated using a visual analog scale (VAS). At follow-up, active headache was defined as a score on the VAS > 3. Multiple linear regressions were used to investigate the associations of migraine at baseline with other PPS at follow-up.

**Results:**

Compared with the migraine with inactive headache group and the non-migraine group, patients with migraine with active headache had significantly higher intensities of other PPS and a lower remission rate of depression. There were no significant differences in the pain intensities of the other seven PPS between the migraine with inactive headache group and the non-migraine group. Headache intensity was significantly correlated with the intensities of other PPS at baseline and follow-up. Migraine with active headache independently predicted other PPS after controlling for depression and anxiety at baseline.

**Conclusions:**

Migraine with active headache among MDD patients could predict other PPS. Prevention and treatment of headache might help to decrease other PPS and improve the prognosis of depression. Integration of treatment for depression and headache is indicated.

## Introduction

Among psychiatric outpatients with mood and/or anxiety disorders, painful physical symptoms (PPS) are common, and are associated with a higher severity of psychopathology [1]. PPS and depression interact closely [2,3]. Among patients with major depressive disorder (MDD), PPS are common [4,5], and are associated with worse depression, an increased suicidal risk, functional impairment, a poorer quality of life, and a poorer treatment response [2–6].

Mood disorders and migraine are closely related [7–10]. Migraine is a common comorbidity among patients with MDD [11–13]. Shared underlying genetically-determined disease mechanisms of migraine and depression have been reported [14,15]. Among patients with MDD, comorbidity with migraine was found to be related to greater severities of depression and anxiety, and had a negative impact on recovery of health-related quality of life post-pharmacotherapy [9,11,12,16]. In a general population, migraine or severe headache are associated with increased other PPS [17]. Among patients with MDD, migraine was also found to be associated with increased other somatic and pain symptoms [12,13]. Moreover, migraine was also associated with an increased suicidal risk [8,18].

As described above, depression, migraine, and PPS interact closely [2,12–14,19,20]. Although previous studies have reported the impacts of migraine on somatic symptoms or PPS among patients with MDD [12,13], these studies were cross- sectional or short-term follow-up studies. One study reported that migraine independently predicted the intensities of eight PPS at baseline and four PPS post-four-week pharmacotherapy among patients with MDD [12]. Moreover, one study focused on the associations of migraine with muscle soreness or pain among patients with MDD, and found that migraine at baseline could independently predict muscle soreness or pain in the upper and lower limbs at the two-year follow-up point [21]. However, the associations of migraine with other pains (such as chest pain, abdominal pain, and joint pain) at the two-year follow-up point among patients with MDD have not been studied in depth. Investigating this issue is important, because PPS have significant negative impacts on MDD [2–6], and understanding the associations of migraine with PPS might help to prevent and treat PPS, in turn improving the prognosis of MDD. Therefore, this study aimed to investigate the associations of migraine with PPS among patients with MDD at the two-year follow-up point. We hypothesized that MDD patients with migraine may still have greater severities of PPS at the two-year follow-up point, because previous studies reported that patients with migraine were associated with increased other PPS [12,13,17].

## Methods

### Subjects

This study was conducted from September 2005 to August 2009 in the psychiatric outpatient clinics of the Chang Gung Memorial Hospital, a medical center in northern Taiwan. The project was approved by the Institutional Review Board of the same hospital. Based on the guidelines regulated in the Declaration of Helsinki, written informed consent was obtained from all participants before enrollment.

At baseline, subjects aged 18–65 years who were experiencing a current major depressive episode (MDE) were enrolled from consecutive outpatients [22]. MDD and MDE were diagnosed by a psychiatrist based on the Structured Clinical Interview for DSM-IV- text revision (TR) Axis I Disorders [23], which is a semi-structured interview created to make reliable psychiatric diagnoses based on the DSM-IV-TR criteria and is designed to be administered by a mental health professional. Four exclusion criteria were established in order to prevent depression and PPS from being confounded: 1) any antidepressant treatment or other psychotropic drug treatment within the previous month; 2) severe psychomotor retardation, psychotic features, or catatonic features; 3) chronic medical diseases such as hypertension, diabetes mellitus, and other medical diseases, except headaches; and 4) a history of substance abuse and/or dependence without full remission in the past month. The second exclusion criteria excluded outpatients with a compromised capacity to consent. Therefore, the enrolled patients with MDD had the capacity to consent.

### Assessment of headaches

A structured headache intake form, based on the operational criteria of the *International Classification of Headache Disorders*, 2nd edition, was used [24]. Questions regarding headache frequency, intensity, pattern, duration, location, precipitating factors, aggravation by physical activities, aura, nausea, vomiting, photophobia, phonophobia, and medication usage for headache were included. After the participants had completed the headache intake form at baseline, an experienced headache specialist, who was blind to the data related to PPS and psychiatric evaluation, interviewed the subjects and made diagnoses. In an analysis of the data, headache diagnoses were updated in accordance with the ICHD-3 beta [25]. Subjects with migraine without aura and/or migraine with aura were classified as the "migraine" group, while the other subjects were classified as the "non-migraine" group.

The frequency and intensity of headache were evaluated both at baseline and at the two-year follow-up point. A visual analog scale (VAS), with 0 representing “no pain” and 10 representing “pain as severe as I can imagine”, was used to evaluate the average headache intensity in the past week. Headache frequency (the number of headache days in the past week) was recorded. At follow-up, a headache intensity VAS score > 3 in the migraine group was considered to indicate active migraine (migraine with active headache), and a VAS score < = 3 indicated inactive migraine (migraine with inactive headache).

### Assessment of other PPS, depression, and anxiety

A (0–10) VAS was used to evaluate the average pain intensities of other PPS in the previous week at baseline and follow-up, including pain in bone and/or joints (bone/joints), back, chest, abdomen, neck and/or shoulder (neck/shoulder), general muscle, and limbs.

The 17-item Hamilton Depression Rating Scale (HAMD) was used to evaluate the severity of depression at baseline and follow-up [26]. Full remission of depression was defined as a HAMD score < = 7. The Hospital Anxiety and Depression Scale (HADS), including a 7-item depression subscale (HADS-depression) and a 7-item anxiety subscale (HADS-anxiety), was used to evaluate the severities of depression and anxiety [27]. The total scores ranged from 0 to 52 for the HAMD and 0 to 21 for the HADS-anxiety and HADS-depression. A higher score indicated a greater severity.

### Procedures

After enrollment, venlafaxine extended-release (one 75 mg capsule per day) and zolpidem were prescribed in the first four weeks. In order to observe the impact of migraine on PPS after acute treatment [12], pharmacotherapy was controlled to avoid confounding from different medications during the four-week period. After this four-week treatment period, pharmacotherapy was not controlled, with the treatment goal of full remission of depression, and the subjects were treated as general psychiatric outpatients. Some patients may have quit pharmacotherapy during the study period. The investigators attempted to contact each subject at the twenty-fourth month after enrollment, which was the index follow-up month. There were four groups at follow-up: 1) subjects who were unable to be contacted by mail and/or phone; 2) subjects who could be contacted and who refused to attend follow-up; 3) subjects who accepted follow-up and who had received pharmacotherapy in the index follow-up month; and 4) subjects who accepted follow-up and who did not undergo pharmacotherapy in the index follow-up month. Only the fourth group was included in the statistical analyses. The third group was excluded because pharmacotherapy without controlling the medications might confound the severities of depression, anxiety, headache and PPS.

### Statistical methods

All statistical analyses were performed using SPSS for Windows 20.0 (SPSS Inc., IBM Corporation, Armonk, NY, USA). The independent *t* test, Mann-Whitney U test, Kruskal-Wallis H test with pairwise comparison, Chi-square test, and Spearman’s correlation were used as appropriate.

Two models of multiple linear regressions with forward selection were used to test the associations of migraine with PPS at the two-year follow-up point. In the first and second models, the dependent variables were the intensities of eight pains at follow-up. In the first model, the independent variables included five demographic variables (age, gender, educational years, employment status, and marital status) at baseline and the presence of migraine or not at baseline. To form the second model, three independent variables were added into the first regression model, including migraine with active headache, HAMD score at baseline, and HADS-anxiety score at baseline. A two-tailed *P* value <0.05 was considered statistically significant. In the two regression models, Bonferroni correction (statistical significance: *P* < 0.00625) was employed.

## Results

### Subjects

At baseline, 155 subjects, who had not received treatment with any antidepressants or psychotropic drugs for at least two months, were enrolled. At the two-year follow-up point, 54 subjects were excluded from further statistical analyses, including 13 who were unable to be contacted, 11 who refused to participate in the follow-up assessment, and 30 who were undergoing pharmacotherapy in the index follow-up month. The remaining 101 subjects, who had discontinued pharmacotherapy and attended follow-up, were included in the statistical analyses (S1 Dataset). The duration from discontinuation of pharmacotherapy to the follow-up point was 17.1 ± 5.8 months among the 101 subjects. Table 1 shows the demographic variables and pain intensities at baseline in the two groups. There were no significant differences at baseline in the five demographic variables, the percentage of patients with migraine, the psychometric scores, or the eight pain intensities between the inclusion and exclusion groups.

**Table 1:** Demographic variables, psychometric scores, and pain intensities in the exclusion and inclusion groups at baseline^a^^,^^b^.

	Exclusion group (*n* = 54)	Inclusion group (*n* = 101)
Age at enrollment	29.9 ± 7.6	30.4 ± 8.2
Educational years	13.0 ± 2.8	13.6 ± 2.3
Women (%)	70.4	67.3
Employment (yes, %)	63.0	57.4
Married (yes, %)	37.0	39.6
Migraine (yes, %)	55.6	42.6
HAMD score	23.7 ± 3.8	23.2 ± 4.1
HADS-depression score	14.6 ± 3.6	14.2 ± 3.3
HADS-anxiety score	15.3 ± 3.1	14.5 ± 3.5
Headache	4.4 ± 3.0	4.1 ± 3.2
Bone/joint pain	2.9 ± 3.0	2.5 ± 3.1
Back pain	3.6 ± 3.3	2.7 ± 3.0
Abdominal pain	1.7 ± 2.4	1.9 ± 2.6
Chest pain	2.4 ± 3.0	2.5 ± 3.2
Neck/shoulder pain	5.0 ± 3.6	5.1 ± 3.3
General muscle pain	2.8 ± 3.2	3.2 ± 3.2
Limb pain	1.2 ± 2.3	1.6 ± 2.6

HAMD = Hamilton Depression Rating Scale; HADS = Hospital Anxiety and Depression Scale.

^a^Pain intensity was evaluated using a 0–10 visual analog scale.

^b^There were no significant differences in any of the data at baseline between the inclusion and exclusion groups.

Among the 101 subjects who were included in the statistical analyses, 43 subjects (42.6%) had migraine, including 1 with chronic migraine and medication overuse headache, 8 with chronic migraine, 3 with episodic migraine both with and without aura, and 31 with episodic migraine without aura. Of the other 58 subjects, 19 subjects had probable migraine without aura, 21 had episodic tension-type headache (TTH), 6 had probable episodic TTH, 6 had unspecified headache, and 6 reported no headache. Compared with the non-migraine group, the migraine group had a greater number of headache days in the past week (4.8 ± 2.4 vs. 2.2 ± 2.2, *P* < 0.01 at baseline; 1.8 ± 2.0 vs. 1.3 ± 1.7, *P* = 0.12 at follow-up). Moreover, the migraine group also included a higher percentage of subjects with active headache at baseline (76.7% vs. 36.2%, *P* < 0.001) and follow-up (41.9% vs. 24.1%, *P* = 0.08) as compared with the non-migraine group.

### Differences in the eight pain intensities between groups

Table 2 shows the differences in the eight measured pain intensities at baseline and follow-up between patients with and without migraine. At baseline, the migraine group had significantly greater pain intensities of the eight pains and higher HAMD scores as compared with the non-migraine group. At follow-up, the migraine group still had significantly higher pain intensities in the head, abdomen, chest, and general muscle as compared with the non-migraine group. No significant differences in the severities of depression and anxiety were noted between the two groups at follow-up.

**Table 2:** Differences in pain intensities and psychometric scores between patients with and without migraine at baseline.

	Pain intensities at baseline		Pain intensities at follow-up	
	Migraine (*n* = 43)	Non-migraine (*n* = 58)	Migraine (*n* = 43)	Non-migraine (*n* = 58)
Headache	6.1 ± 2.9**	2.6 ± 2.5	2.8 ± 2.8*	1.8 ± 2.4
Bone/joint pain	3.8 ± 3.4**	1.4 ± 2.3	2.0 ± 2.9	1.3 ± 2.2
Back pain	4.1 ± 3.3**	1.6 ± 2.3	2.3 ± 2.9	1.6 ± 2.4
Abdominal pain	2.8 ± 2.8**	1.3 ± 2.2	1.4 ± 2.3*	0.7 ± 1.7
Chest pain	3.5 ± 3.7**	1.8 ± 2.6	1.5 ± 2.6**	0.5 ± 1.4
Neck/shoulder pain	6.3 ± 3.1**	4.2 ± 3.1	3.5 ± 3.5	3.0 ± 2.8
General muscle pain	4.5 ± 3.3**	2.2 ± 2.8	2.4 ± 3.3*	1.1 ± 2.1
Limb pain	2.4 ± 3.0**	0.9 ± 2.0	1.4 ± 2.6	0.8 ± 1.8
HAMD score	24.4 ± 4.3*	22.3 ± 3.6	10.7 ± 8.1	9.3 ± 7.3
HADS-depression score	14.9 ± 3.0	13.7 ± 3.5	7.2 ± 4.8	6.0 ± 5.2
HADS-anxiety score	15.2 ± 3.5	14.0 ± 3.5	9.1 ± 4.3	8.0 ± 4.6

HAMD = Hamilton Depression Rating Scale; HADS = Hospital Anxiety and Depression Scale.

* *P* < 0.05

** *P* < 0.01

Table 3 shows the differences in the eight pain intensities in the three groups (including migraine with active headache, migraine with inactive headache, and non-migraine) at follow-up. The migraine with active headache group had significantly greater pain intensities of all pains except abdominal pain, and higher HAMD scores, than the migraine with inactive headache and the non-migraine groups. There were no significant differences in all pain intensities, depression, and anxiety between the migraine with inactive headache group and the non-migraine group.

**Table 3:** Differences in pain intensities and psychometric scores at the two-year follow-up point between the three groups^a^.

	Migraine with active headache (*n* = 18)	Migraine with inactive headache (*n* = 25)	Non-migraine (*n* = 58)
Headache	5.5 ± 2.1	0.9 ± 1.0***	1.8 ± 2.4^###^
Bone/joint pain	3.8 ± 3.3	0.7 ± 1.8***	1.3 ± 2.2^##^
Back pain	4.4 ± 3.2	0.8 ± 1.5***	1.6 ± 2.4^##^
Abdominal pain	2.4 ± 3.0	0.7 ± 1.3	0.7 ± 1.7^#^
Chest pain	2.9 ± 3.3	0.4 ± 1.2**	0.5 ± 1.4^###^
Neck/shoulder pain	5.6 ± 3.6	2.0 ± 2.9**	3.0 ± 2.8^#^
General muscle pain	4.4 ± 3.6	1.0 ± 2.1**	1.1 ± 2.1^###^
Limb pain	2.7 ± 3.3	0.4 ± 1.1**	0.8 ± 1.8^#^
HAMD score	15.7 ± 8.6	7.0 ± 5.6**	9.3 ± 7.3^#^
HADS-depression score	8.8 ± 4.2	6.0 ± 5.0	6.0 ± 5.2^#^
HADS-anxiety score	10.8 ± 4.0	7.8 ± 4.1	8.0 ± 4.6

HAMD = Hamilton Depression Rating Scale; HADS = Hospital Anxiety and Depression Scale.

* *P* < 0.05

** *P* < 0.01

*** *P* < 0.001 in the migraine with active headache group *vs*. the migraine with inactive headache group.

^#^ *P* < 0.05

^##^ *P* < 0.01

^###^ *P* < 0.001 in the migraine with active headache group *vs*. the non-migraine group.

^a^There were no significant differences in all values between the migraine with inactive headache group and the non-migraine group.

The full remission rates of depression differed significantly (*P* < 0.01) between the three groups (migraine with active headache, 16.7%; migraine with inactive headache, 68.0%; non-migraine, 39.7%). In the non-migraine group, the subjects with active headache (2/14) also had a significantly lower remission rate of depression (14.3% *vs*. 47.7%, *P* = 0.02) than the subjects with inactive headache (21/44).

### Correlations of headache intensity with intensities of other PPS

At baseline, headache intensity was significantly correlated with the other seven PPS, with correlation coefficients (*r*) ranging from 0.50 to 0.30 (all *P* < 0.01). At follow-up, headache intensity and the other seven PPS were also significantly correlated (*r*: 0.40 to 0.25, all *P* < 0.05).

### Migraine independently predicted other pains at follow-up

Migraine at baseline was not identified in the first regression model as a significant factor predictive of the intensities of all PPS at follow-up after Bonferroni correction. In the second regression model (Table 4), migraine with active headache was the most significant factor predicting the intensities of the eight pains at follow-up when controlling for demographic variables and the severities of depression and anxiety at baseline and after Bonferroni correction.

**Table 4:** Independent variables predicting different pains at the two-year follow-up point^a^^,^^b^^,^^c^.

Dependent variable	Independent variable	Beta	*R*^*2*^ change	*t*	*P*
Headache	Active migraine	0.55	0.33	6.76	< 0.001
	Educational years	−0.16	0.03	−2.00	0.049
Bone/joint pain	Active migraine	0.37	0.15	4.08	< 0.001
	HADS-anxiety	0.24	0.06	2.67	< 0.001
Back pain	Active migraine	0.44	0.20	4.90	< 0.001
Abdominal pain	Active migraine	0.32	0.10	3.36	0.001
Chest pain	Active migraine	0.38	0.21	4.08	< 0.001
	HAMD	0.24	0.05	2.64	0.009
Neck/shoulder pain	Active migraine	0.36	0.13	3.81	< 0.001
General muscle pain	Active migraine	0.41	0.23	4.43	< 0.001
	HAMD	0.20	0.04	2.20	0.03
Limb pain	Active migraine	0.27	0.13	2.80	0.006
	HAMD	0.24	0.05	2.48	0.015

HAMD = Hamilton Depression Rating Scale; HADS = Hospital Anxiety and Depression Scale.

^a^HAMD and HADS-anxiety were evaluated at baseline.

^b^Active migraine represented patients with migraine at baseline who had active headache at the two-year follow-up point.

^c^Multiple linear regressions with the forward method were employed.

Based on the second regression model, if the sample size was extended to include the patients in group 3 (patients with pharmacotherapy in the index month; n = 30) and group 4 (patients without pharmacotherapy in the index month; n = 101), and pharmacotherapy (yes or no) was added as an independent variable, the results showed that active migraine was still the most significant factor associated with the eight PPS after controlling for demographic variables, depression, and anxiety. Pharmacotherapy was not a significant factor.

## Discussion

The second regression model demonstrated that migraine with active headache was a significant factor related to all of the eight pains assessed at follow-up after controlling for the severities of depression and anxiety. However, the first regression model showed that migraine at baseline was not significantly associated with PPS at follow-up. The results presented in Table 3 show that the migraine with active headache group had significantly higher intensities of pains than the migraine with inactive headache group and the non-migraine group. Conversely, there were no significant differences between the migraine with inactive headache group and the non-migraine group. These results demonstrated that the association of migraine with other PPS only occurred in subjects with migraine with active headache. This might be the reason for which migraine was not identified as a significant factor related to other PPS in the first regression model. One previous study also demonstrated no significant differences in depression, anxiety, and somatic symptoms between patients with inactive migraine and patients without migraine among patients with MDD [28].

These results had clinical implications. First, active headache might be an important index to predict other PPS among MDD patients with migraine. When headache occurs, there may be a greater probability of the appearance of other PPS. Our results showed that headache intensity was significantly correlated with the intensities of the other 7 pains, both at baseline and follow-up. In clinical practice, physicians should assess the severity of headache. Second, according to previous studies, PPS are associated with a poorer quality of life and a poorer treatment outcome of depression [2,4,6]. Moreover, our results showed that patients with migraine with active headache had the lowest remission rate of depression, as compared with patients with migraine with inactive headache and non-migraine subjects. There is a possibility that prevention of headache might decrease the intensities of other PPS, subsequently improving the outcome of depression. In fact, active headache was found to be a significant factor related to a lower full remission rate of depression among patients with MDD [28].

Migraine with active headache was found to be associated with other PPS. This might be a result of repeated headache attacks among patients with migraine possibly leading to central sensitization, which manifests as hyperalgesia, allodynia, and spontaneous pain [29–31]. In fact, sensory hypersensitivities and somatosensory amplification are common phenomena in cases of migraine [31–33]. Therefore, MDD patients with migraine and active headache might become more sensitive to other PPS. Previous studies have shown that depression, anxiety, migraine, allodynia, and PPS are closely related [34–36].

Some methodological issues or limitations should be noted. 1) This study did not include patients undergoing pharmacotherapy at follow-up, because pharmacotherapy might confound the severities of headache and other PPS. However, patients with or without pharmacotherapy were not divided by randomization, but by patients’ decisions. Possible bias could not be excluded. 2) This study aimed to examine whether variables at baseline could predict PPS at follow-up. Therefore, independent variables included demographic variables, migraine, HAMD score, and HADS-anxiety score at baseline. 3) This study was carried out in a medical center. Expansion of these results to a general population should be performed with caution. 4) Some of the eight pains might overlap; for example, general muscle pain might overlap with pain in other areas. 5) At baseline, a headache diary was not used because withholding pharmacotherapy while prospectively observing headache parameters might result in ethical problems owing to suicidal risk. At follow-up, the headache parameters in the past week were recalled by the patients and compared with the baseline data, which might have introduced recall bias. 6) Future studies should investigate the associations of treatment responses of PPS during acute treatment with the long-term (two-year) outcomes of PPS.

## Conclusion

Migraine with active headache was independently associated with other PPS at the two-year follow-up point. Compared with the migraine with inactive headache group and the non-migraine group, the patients with migraine with active headache had significantly higher pain intensities of other PPS and a lower remission rate of depression at follow-up. Conversely, there were no significant differences in the pain intensities of other PPS between the migraine with inactive headache group and the non-migraine group. Prevention and treatment of headache might be helpful in terms of decreasing other PPS, which may in turn improve the prognosis of MDD. Integration of treatment for depression and headache is indicated.

## Supporting information

S1 Dataset The dataset includes demographic variables, scores of psychometric scales, headache indices, and different pain intensities at baseline and 2-year follow-up. (XLSX)
